# Patient specific quality assurance for the delivery of intensity modulated radiotherapy

**DOI:** 10.1120/jacmp.v4i1.2540

**Published:** 2003-01-01

**Authors:** Nzhde Agazaryan, Timothy D. Solberg, John J. DeMarco

**Affiliations:** ^1^ Department of Radiation Oncology UCLA School of Medicine 200 UCLA Medical Plaza, Suite B265 Los Angeles California 90095‐6951

**Keywords:** IMRT, dosimetry, treatment verification, quality assurance

## Abstract

A patient specific quality assurance program has been developed to facilitate the clinical implementation of intensity modulated radiotherapy (IMRT) delivered using a micro‐multileaf collimator. The methodology includes several dosimetric tasks that are performed prior to the treatment of each patient. Film dosimetry is performed for each individual field and for the multifield composite plan. Individual field measurements are performed at a depth of 5 cm in a water equivalent slab phantom; export of dose calculations from the treatment planning system is similarly specified. For the composite distribution, parameters from the patient plan are applied to an IMRT phantom, and film is exposed in an axial orientation. Distributions are compared with the aid of software developed for the specific tasks. The measured and calculated dose distributions can be superimposed and positioned graphically using move, rotate, and mirror tools, as well as by specifying isocenter coordinates and using fiducial marks. Horizontal and vertical profiles are available for analysis. Dose difference, distance‐to‐agreement, and *γ* index, the minimum scaled multidimensional distance between a measurement and a calculation point determined in combined dose and physical distance space, are calculated along a specified isodose line and displayed. *γ* provides an excellent measure of disagreement between measurement and calculation for complex intensity distributions. We specify 3% dose difference and 3 mm distance as our scaling acceptability criteria. Absolute dosimetry for each composite plan is performed using an ionization chamber. To date, excellent agreement between measurements and calculations has been observed. © *2003 American College of Medical Physics*.

PACS number(s): 87.53.–j, 87.66.–a

## I. INTRODUCTION

The concept of conforming the dose in three‐dimensions is not new; the use of compensators, wedges, and dynamic asymmetric jaws is targeted toward that process. Intensity modulated radiotherapy (IMRT), being conformal radiotherapy, has the same goal of conforming the physical dose in three dimensions. The advancement of conformal therapy is one of the most promising developments to take place in radiotherapy during the past decade due to development of computer applications in diagnostic imaging and radiotherapy. A number of studies have demonstrated the superiority of the physical dose distribution of IMRT compared to other modalities, with applications in brain tumors, head and neck cancers, and prostate cancer treatments.^1^‐^5^ The reason and rationale for the effort put in this area is to reduce the risk or severity of complications where radiotherapy is successful, and to escalate the dose while reducing or keeping a comparable level of complications, where the failure of radiation therapy is the lack of local control.^6^‐^10^

Conventional devices, such as wedges and compensators, modulate the intensity of a beam, and subsequently the energy fluence, such that all points in a field are continuously irradiated, hence the term *spatial modulation*. Another method of varying the energy fluence distribution across the field is through *temporal modulation*. During this process, the energy fluence modulation is achieved not by modulating the intensity of the beam across the field, but rather by modulating the time that each subpart of a field is exposed to radiation. An example of this is a process called dynamic wedging, during which the jaw moves uniformly while the radiation beam is on, producing a wedge‐shaped dose distribution^11^‐^13^ Multileaf collimator (MLC) intensity modulated radiation therapy is an advanced form of conformal therapy. With the use of a multileaf collimator, the exposure time of different sections of the field to primary radiation is modulated in two dimensions, resulting in two dimensional energy fluence modulations across the field.

Dosimetric accuracy requirements have been developed for “conventional” treatments.^14^ Dose distributions are analyzed based on dose gradients. Low dose gradient regions are required to meet the acceptance criteria placed on dose difference, and high dose gradient regions are required to meet the acceptance criteria placed on distance‐to‐agreement (DTA). Tolerance levels for photon beam calculations in homogeneous media are 3% and 4 mm, respectively. While techniques to calculate and deliver IMRT are presently reaching a level of maturity within the academic and clinical communities, methods for direct verification of the delivery, as well as definitions of acceptability of a treatment in terms of these measurements, are the most problematic at this stage of IMRT advancement.

Patient specific dosimetric verification of an IMRT plan is an important part of clinical implementation of IMRT into any clinic. A number of IMRT quality assurance (QA) studies have been published recently that address the issue of IMRT QA and in some cases patient specific QA.^14^‐^22^ The approach taken at our institution shares some similarities with the above‐mentioned publications, however, the approach has many differences in terms of methodology, equipment, measurements, and especially methods of measurement analysis.

## II. MATERIALS AND METHODS

### A. IMRT software and hardware

The measurements presented have been conducted with the use of the Novalis® linear accelerator (BrainLAB, AG, Heimstetten, Germany). The underlying accelerator is a Varian Clinac® 600SR unit (Varian Associates, Palo Alto, CA), a C‐Series linear accelerator generating a 6 MV x‐ray beam. The dose rate is variable in four equal increments of 160 MU/min. The minimum dose rate is 160 MU/min and maximum dose rate is 800 MU/min. The dose rate for IMRT treatments is set to 480 MU/min.

The Novalis® is equipped with an $m_{3}$™ (BrainLAB, AG, Heimstetten, Germany) micro‐multileaf collimator (mMLC). The mMLC is an accessory for Varian C‐Series radiotherapy machines.

The $m_{3}$™ micro‐MLC consists of fifty‐two tungsten leaves that shape a treatment field of up to $10\times 10{\;\text{cm}}^{2}$.^23^ The leaf widths range from 3.0 mm at the center of the field to 5.5 mm at the periphery. The MLC consists of fourteen 3.0 mm wide, six 4.5 mm wide, and six 5.5 mm wide leaf pairs. The front faces of the leaves are shaped to minimize the penumbra variations as a function of position. Maximum distance over centerline is 5.0 cm, maximum retract distance is 5.0 cm, and maximum leaf spread is 10.0 cm. The specification for maximum leaf speed is 1.5 cm/s and the specification for the leaf positioning accuracy is better than 0.1 mm. The system is capable of dynamic and segmented IMRT, however, only segmented IMRT is used clinically at our institution.

IMRT plans presented have been generated using commercially available IMRT treatment planning system (BrainSCAN® version 5, BrainLAB AG, Heimstetten, Germany). This micro‐MLC based intensity modulated radiation surgery (IMRS) system is BrainLAB's high‐resolution version of IMRT. The inverse planning algorithm is based on the dynamically penalized likelihood (DPL) algorithm. The algorithm itself is based on a maximum likelihood estimator (MLE) method of statistical parameter estimation used initially in image reconstruction. The relationship between the MLE and DPL is described in detail by Llacer.^24^ The target function used in the algorithm is adapted from a PET image reconstruction algorithm developed by Shepp and Vardi.^25^,^26^ The algorithm is robust in that it will always produce reasonable results.

### B. Dry Water™ Slab and MED‐TEC IMRT phantom dosimetry

**Figure 1:**
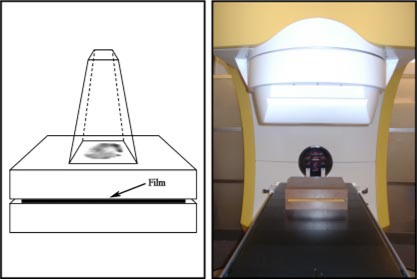
(Color) (Left) Schematic diagram of the experimental setups of Radiology Support Devices (RSD) Dry Water™ Slab phantom used for single field IMRT measurements. (Right) A picture of a single field IMRT measurement setup.

**Figure 2:**
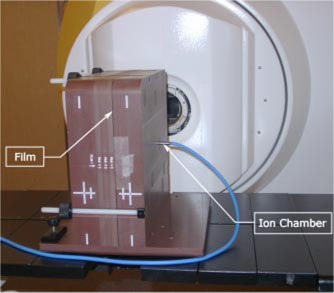
(Color) The MED‐TEC IMRT phantom made of Virtual Water™ during the absolute dose measurement.

Film dosimetry has been used in this study. Film measurements have been conducted with a Radiology Support Devices (RSD) Dry Water™ Slab phantom (RSD, Long Beach, CA), the experimental setup of which is shown in Fig. 1, and a MED‐TEC IMRT phantom made from Virtual Water™ (MED‐TEC, Orange City, IA) shown in Fig. 2. Dry Water™ and Virtual Water™ phantoms meet International Commission on Radiation Units and Measurements Report 44 (ICRU 44) guidelines.^27^ Film dosimetry is a standard method of obtaining two‐dimensional dose distributions.^28^‐^30^ Because the accuracy and precision of the film measurements are dependent on measurement conditions and processing, film dosimetry is not a reliable method of absolute measurements, however, it is a valuable tool for relative measurements and periodic quality assurance measurements.^31^

IMRT patient specific QA requires a method of absolute dose measurements. The ionization chamber is the standard dosimeter for calibration and absolute dose measurement for radiation therapy.^32^,^33^ As such, a 0.125cc ionization chamber (Semiflex Model 31002, PTW, Freiburg, Germany) has been used for absolute dose measurements in the MED‐TEC IMRT phantom.

### C. A software tool for quantitative comparative analysis

A software tool has been developed to perform quantitative comparative analysis of two datasets. This in‐house software is written in a development language (IDL, Research Systems, Inc., Boulder, CO) and runs on Microsoft® Windows® (Microsoft Corp., Redmond, WA) environment. The tool is used as a platform for comparative analysis of the measured and calculated dose distributions. The measured and calculated dose distribution maps can be superimposed and positioned manually using move, rotate, and mirror tools. Absolute positioning is also available by specifying the isocenter coordinates from a treatment planning dataset and specifying two pairs of marks on the film, defining two lines, the crossing point of which defines the isocenter on the film. Dose difference, distance‐to‐agreement, and γ index introduced by Low *et al.* along any specified isodose line can be displayed.^34^ Horizontal and vertical profiles of these quantities through any specified point of the dose distribution map and two‐dimensional map of the γ index are available. The γ index is the minimum scaled multidimensional distance between a measurement and a calculation point, determined in combined dose and physical distance space. The scaling parameters of dose difference and distance are user inputs. For clinical patient specific QA we specify 3% dose difference and 3 mm distance acceptance scaling criteria.^14^ The magnitude of γ provides a measure of disagreement between measurement and calculation. Regions where γ is larger than unity correspond to locations where the calculation does not meet the acceptance criteria. Analysis of the single field IMRT measurements using a Dry Water™ Slab phantom and the composite plan measurement using a MED‐TEC IMRT phantom are performed with the help of the software. All the results of the analysis of the data are printed and/or saved in a postscript file.

### D. Patient specific quality assurance protocol

We have developed a patient specific QA protocol for IMRT patient pretreatment verification. The protocol consists of two parts: absolute dosimetry and relative dosimetry. The film measurements are primarily concerned with investigating the relative dosimetric agreement between the planned and measured dose distributions. They provide important information about overall distribution in a particular plane. In addition, ion chamber verification provides information about absolute agreement at a point.

The protocol developed for IMRT patient treatment verification is as follows. All fields are individually delivered and measured in the Dry Water™ Slab phantom with an SAD setup shown in Fig. 1 at 5.0 cm depth. For individual fields Kodak XV2 (Eastman Kodak, Rochester, NY) film is used and monitor units (MU) are scaled to avoid film saturation. Monitor unit scaling does not change the dynamics of the MLC movements, since the clinical cases are being delivered using segmented IMRT. In addition, the composite plan is delivered onto the MED‐TEC IMRT phantom shown in Fig. 2 and axial dose distribution is measured using Kodak EDR2 (Eastman Kodak, Rochester, NY) film, where the isocenter is positioned at the film plane. The composite film measurement is in the plane of the leaf movements and is largely affected only by the leaf pairs corresponding to that plane and neighboring pairs. Besides being more sensitive measurements, the single field measurements provide QA for all the leaf pairs. Additionally, absolute dosimetry for each composite plan is performed using an ionization chamber, with the ion chamber positioned at isocenter. The center of the ion chamber sensitive volume is located 3.8 cm lateral from the center of the most superior MED‐TEC IMRT phantom slab.

Pixel gray scale value to dose conversion of the XV2 and EDR2 film data is performed using the sensitometric curves obtained from calibration films. Calibration films are obtained by exposing a series of XV2 and EDR2 films to 10 by 10 cm^2^ fields at 1.5 cm depth and 98.5 cm source to surface distance (SSD) in Dry Water™ over the useful range of each type of film. The calibration technique ignores any depth variation of the film response, which has been shown to be minimal for up to field sizes of 10 by 10 cm^2^.^35^

The film measurements are compared with the planned dose distribution using the described in‐house software. The treatment planning system has an option of exporting dose distribution at specified depth assuming a cubic water phantom, allowing for IMRT single field dose measurement and calculation comparisons.

**Figure 3:**
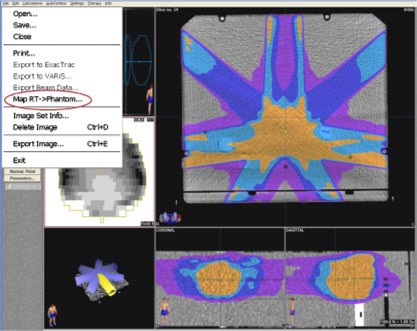
(Color) The treatment planning system has an option of mapping the complete patient treatment onto any phantom. A seven field prostate IMRT plan is shown mapped onto the MED‐TEC IMRT phantom.

The MED‐TEC IMRT phantom, made of Virtual Water™ and shown in Fig. 2, is computed tomography (CT) compatible with visible fiducial markers. The phantom is scanned on a CT scanner and the images are transferred to the treatment planning system. The treatment planning system has an option of mapping the complete patient treatment onto any phantom (Fig. 3). After mapping the patient treatment plan onto the MED‐TEC phantom, the dose distribution in the phantom can be viewed and exported. The exported distribution is then compared to the measured distribution with our in‐house software discussed previously. The MED‐TEC IMRT Phantom became an essential part of quality assurance at our institution.

**Figure 4:**
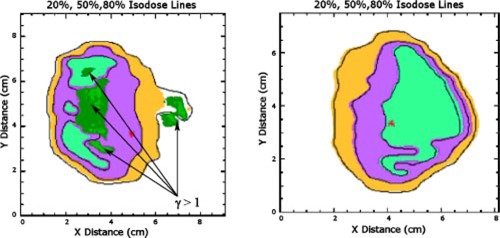
(Color) The 20%, 50%, and 80% isodose lines of calculation and measurement are shown. Measured data is in the form of color wash, and calculated data is presented in terms of solid lines. The γ index distribution is superimposed on the calculated and measured dose maps. For the regions where the γ index is larger than unity, the program outputs a map of γ index with different intensities of green corresponding to different magnitudes of γ. Values of 3% and 3 mm have been used for dose difference and distance tolerances respectively. (Left) An example of a QA analysis with significant difference between measurement and calculation. (Right) An example of a QA analysis with no points on the map with γ index larger than unity.

**Figure 5:**
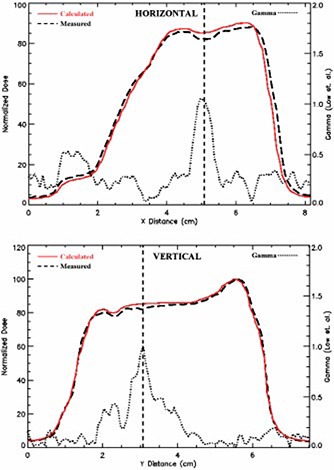
(Color) (Top) Horizontal and (bottom) vertical profiles of measured and calculated dose distribution along with the γ index. The example shown is from the data presented in right.

**Figure 6:**
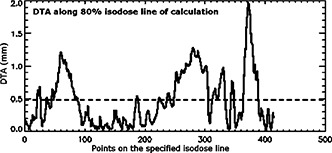
DTA values along the specified (80%) isodose line of the calculation data. The average DTA is less than 0.5 mm and the maximum DTA is less than 2.0 min.

**Figure 7:**
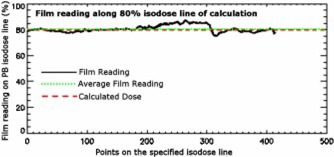
(Color) Film reading along the specified (80%) isodose line of the calculation data.

**Figure 8:**
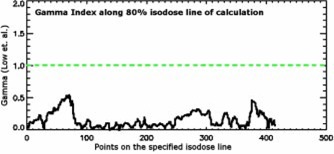
(Color) Gamma index values along the specified (80%) isodose line of the calculation data.

In Figs. 4–8, sample analysis of single field measurements are shown. The program outputs the 20%, 50%, and 80% isodose lines of calculation and measurement, as shown in Fig. 4. Measured data is in the form of color wash, and calculated data is presented in terms of solid lines. The γ index distribution is superimposed on the calculated and measured dose maps. For the regions where the γ index is larger than unity, the program outputs a map of the γ index with different intensities of green corresponding to different magnitudes of γ. Values of 3% and 3 mm are used for dose difference and distance tolerances, respectively. Horizontal and vertical profiles of measured and calculated data, along with the γ index, at any point of the dose distribution are interactively available (Fig. 5). DTA values along any specified isodose line of the calculation data can be displayed as shown in Fig. 6. Film readings (Fig. 7), as well as γ values (Fig. 8), along the same specified isodose line of the calculation data are also displayed, printed, and/or saved in a postscript file.

## III. RESULTS AND DISCUSSION

The analysis tool described is currently being used for IMRT patient specific verification at the UCLA Radiation Oncology department and has proven to be a valuable tool for analyzing the single field measurements using a Dry Water™ Slab phantom and the composite plan measurements using a MED‐TEC IMRT phantom. In addition, other researchers at our institution use this software to compare datasets. In one study, the tool is being used to compare brachytherapy Monte Carlo calculation with TG‐43 based calculations. In another study, the tool is being used to compare IMRT measurements with and without respiratory gating.

**Figure 9:**
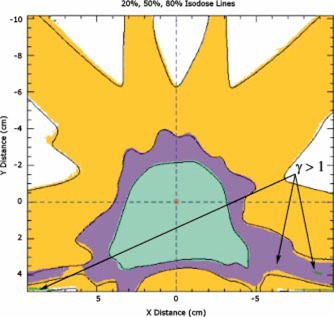
(Color) 20%, 50%, and 80% isodose lines of calculation and measurement of a composite seven field IMRT plan. Measured data is in the form of color wash, and calculated data is in terms of solid lines. The γ index distribution is superimposed on the map using different intensities of green for different magnitudes of gamma.

A comparison of the single field IMRT measurement and calculation as well as comparison of composite plan film measurement with calculation has been performed for every patient treated with IMRT. In general, larger dose difference regions correspond to smaller DTA value regions and vice versa. This is expected, since larger dose differences are expected at high dose gradient regions, where the involved distances are small. An example of a typical composite IMRT film measurement analysis is shown in Fig. 9.

**Figure 10:**
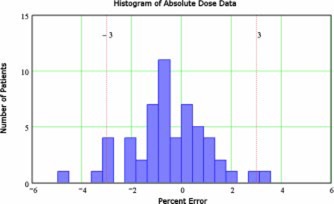
(Color) The histogram of the measured and calculated absolute dose differences for all the patients since establishing the QA protocol.

The agreement between measurement and calculation of composite plan absolute dose has been performed with an ion chamber since the sixth patient treated with IMRT. For only three of these patients the ion chamber has not been positioned at the isocenter since the isocenter position was near the edge of the planning target volume (PTV). The summary of the measured and calculated absolute dose differences for all patients is presented in Table I. The histogram of this data is shown in Fig. 10. The largest error measured so far has been −4.79%, with a mean of only −0.54% and standard deviation of 1.54%. Considering that the reported uncertainty of ion chamber calibration itself is 1.2%, the absolute measurement data is well within the acceptable range. The distribution is very symmetric with a skewness of only 0.07. The data is leptokurtic with a kurtosis of 0.6. The latter means that the center peak around the mean of the distribution is higher than that for a normal distribution.

According to our institutional standard of ±3% acceptability criteria, the treatment plan with the measured error of −4.79% was not acceptable for treatment until it was investigated further and found that the disagreement occurred due to the volume averaging effect of a finite‐size ion chamber. This was a treatment plan with unusually large intensity modulations in the direction perpendicular to leaf motion. Although the cumulative dose distribution did not exhibit a large dose gradient at the isocenter, the dose distributions from individual fields did. The above‐mentioned fact has been identified for several treatment plans with a large disagreement between calculated and measured absolute doses. For these cases, measurements at less intensity modulated regions yielded better results.

**Table I:** The statistical analysis of the percent difference between the calculated and measured absolute doses for all the patients since establishing the QA protocol.

Descriptive statistic for absolute dosimetry data (Patients 6–60)	
**Mean**	−0.54
Standard Error	0.21
Median	−0.59
Mode	−0.71
Standard Deviation	1.54
Sample Variance	2.37
Kurtosis	0.67
Skewness	−0.07
Range	8.32
**Minimum**	−4.79
**Maximum**	**3.53**
Count	55
Number of Negatives	35
Number of Positives	20
Negative to Positive Ratio	1.75
**Confidence Level (95.0%)**	**0.42**

The negative to positive ratio of the absolute dose data is 1.75, and the mean dose difference is a negative number (Table I). We suspect that the reason for the above two facts is volume averaging effect of an ion chamber. During IMRT treatment there are instances where the ion chamber is partially irradiated with primary beam and the displayed reading is a volume‐averaged reading, giving a slightly lower reading.

The dosimetric verification protocol and the software tools used for the analysis of the data discussed are shown to be highly practical. The measurements and the analysis demonstrate that the complete IMRT system at use is accurate and acceptable for patient treatments.

## ACKNOWLEDGMENTS

The authors would like to thank Wolfgang Ullrich and Claus Promberger of BrainLAB (Heimstetten, Germany) for their valuable suggestions and for their help with developing a software tool to perform quantitative comparative analysis.
